# Unfavorable switching of skewed X chromosome inactivation leads to Menkes disease in a female infant

**DOI:** 10.1038/s41598-023-50668-2

**Published:** 2024-01-03

**Authors:** Ayumi Matsumoto, Shintaro Kano, Natsumi Kobayashi, Mitsuru Matsuki, Rieko Furukawa, Hirokazu Yamagishi, Hiroki Yoshinari, Waka Nakata, Hiroko Wakabayashi, Hidetoshi Tsuda, Kazuhisa Watanabe, Hironori Takahashi, Takanori Yamagata, Takayoshi Matsumura, Hitoshi Osaka, Harushi Mori, Sadahiko Iwamoto

**Affiliations:** 1 Division of Human Genetics, Center for Molecular Medicine, Jichi Medical University, Shimotsuke, Tochigi, Japan; 2 Department of Pediatrics, Jichi Medical University, Shimotsuke, Tochigi, Japan; 3 Department of Radiology, Jichi Medical University, Shimotsuke, Tochigi, Japan; 4 Department of Obstetrics and Gynecology, Jichi Medical University, Shimotsuke, Tochigi Japan; 5 Division of Cardiovascular Medicine, Department of Medicine, Jichi Medical University, Shimotsuke, Tochigi Japan

**Keywords:** Diseases, Endocrine system and metabolic diseases, Neurological disorders, Genetics, Clinical genetics, Epigenetics

## Abstract

Menkes disease is an X-linked disorder of copper metabolism caused by mutations in the *ATP7A* gene, and female carriers are usually asymptomatic. We describe a 7-month-old female patient with severe intellectual disability, epilepsy, and low levels of serum copper and ceruloplasmin. While heterozygous deletion of exons 16 and 17 of the *ATP7A* gene was detected in the proband, her mother, and her grandmother, only the proband suffered from Menkes disease clinically. Intriguingly, X chromosome inactivation (XCI) analysis demonstrated that the grandmother and the mother showed skewing of XCI toward the allele with the *ATP7A* deletion and that the proband had extremely skewed XCI toward the normal allele, resulting in exclusive expression of the pathogenic *ATP7A* mRNA transcripts. Expression bias analysis and recombination mapping of the X chromosome by the combination of whole genome and RNA sequencing demonstrated that meiotic recombination occurred at Xp21-p22 and Xq26-q28. Assuming that a genetic factor on the X chromosome enhanced or suppressed XCI of its allele, the factor must be on either of the two distal regions derived from her grandfather. Although we were unable to fully uncover the molecular mechanism, we concluded that unfavorable switching of skewed XCI caused Menkes disease in the proband.

## Introduction

Menkes disease is an X-linked recessive disease caused by mutations in the copper transporter gene *ATP7A* at Xq21.1, which result in the deficiency of the P-type ATPase and reduced activity of various copper-containing enzymes^1^. Severe neurologic disorders begin to develop around 2 to 3 months of age. Impaired activity of lysyl oxidase causes malformation of collagen and elastin and presents with characteristic clinical manifestations, including intellectual disability, epilepsy, subdural hematoma, bone abnormality, and bladder diverticula^2,3^. Most patients are males, and females are rarely affected except for subtle hair and skin abnormalities^4^.

One of the two X chromosomes in female cells is inactivated for gene dosage compensation between XY males and XX females. The process known as X-chromosome inactivation (XCI) is generally random in each cell^5–7^. Because the maternal and paternal X chromosomes have an equal chance of inactivation, most carrier females are spared X-linked recessive diseases. However, with XCI skewing toward the normal allele, that is, when the X chromosome carrying the normal allele is selectively inactivated, female heterozygotes can suffer from the disease due to exclusive expression of the disease allele.

Approximately 8.8% of phenotypically normal females show relatively biased XCI (< 20:80 or > 80:20) and 0.8% show markedly biased XCI (< 5:95 or > 95:5)^8^. On the other hand, pedigrees with multiple females with XCI skewing were also reported, suggesting that XCI may not be completely random, but is modified by a heritable genetic factor^9,10^. Some cases were proposed to be due to genetic variations of the X inactivation center (XIC), the key region to control the XCI process^11^, or cell growth selection against deleterious alleles^12^. For other cases, whether and how XCI skewing is inherited is not fully understood.

We describe here a 7-month-old female patient with Menkes disease caused by partial deletion of the *ATP7A* gene inherited from her mother and her grandmother. While all of them also showed skewing of XCI, only the proband had skewed XCI toward the normal allele. We conducted expression bias analysis and meiotic recombination mapping in an attempt to dissect the underlying mechanism of XCI skewing in this family.

## Results

### Clinical characteristics of the patient

**Figure 1:**
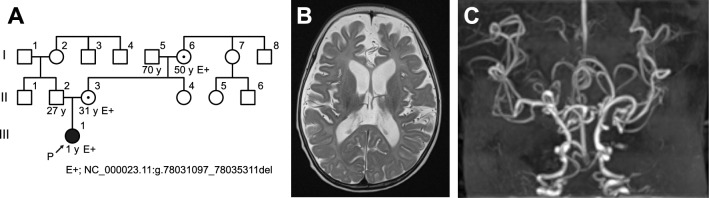
Clinical characteristics of the patient. (**A**) A family tree. Samples from individuals I-5, I-6, II-2, II-3, and III-1 were collected. (**B**) T2-weighted MR imaging showed diffuse atrophy of the cerebrum and delayed myelination in the splenium of the corpus callosum. (**C**) MR angiography showing intracranial tortuous arteries characteristic of Menkes disease.

The proband was a 7-month-old female. She was the first child born to non-consanguineous, healthy parents without a history of genetic disorders, and was born by spontaneous delivery at 37 weeks and 2 days, after infertility treatment (Fig. 1A). At birth, her height was 46.0 cm [− 0.72 standard deviations (SD)], body weight was 2444 g (− 0.46 SD), and occipitofrontal circumference was 34.0 cm (− 1.25 SD). She could not control her head at 7 months of age and was referred to our hospital with the first episode of seizure and delayed development. She had no history of bone fractures or urinary tract infections. On physical examination, we observed hypotonia, pale and lax skin, hypopigmented and sparse hair, and joint laxity, but not kinky hair. Electroencephalography detected the hypsarrhythmia pattern, which indicated West syndrome. Laboratory examination showed lactic acid at 41.8 mg/dL (normal: 3.7–16.3 mg/dL) and pyruvic acid at 1.9 mg/dL (normal: 0.3–0.9 mg/dL). Abdominal ultrasonography showed no abnormalities in the kidneys, ureters, urinary bladder, or other organs. Head magnetic resonance (MR) imaging showed diffuse brain atrophy of the cerebrum, cerebellum, and brain stem. T2-weighted images showed delayed myelination (Fig. 1B). MR angiography demonstrated the tortuous intracranial arteries characteristic of Menkes disease (Fig. 1C). Serum copper and ceruloplasmin levels were subsequently examined, which were low at 15 mg/dL (normal: 25–150 mg/dL) and 3 mg/dL (normal: 15–40 mg/dL). Her karyotype was 46,XX.

### Identification of inherited partial deletion of the *ATP7A* gene

**Figure 2:**
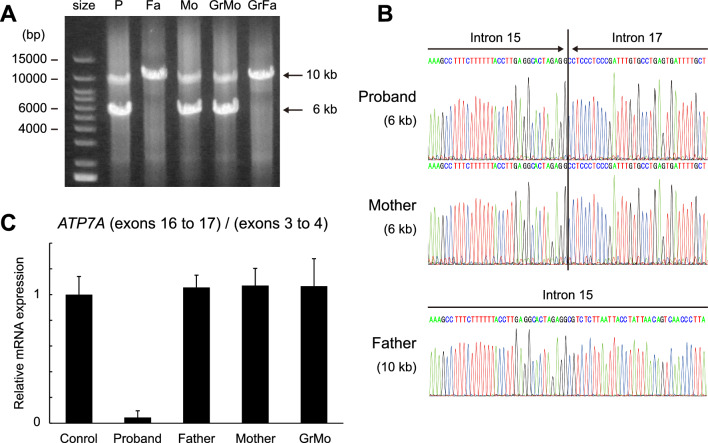
Identification of inherited deletion of exons 16 and 17 of the *ATP7A* gene. (**A**) PCR amplification of exons 15 to 18 of *ATP7A* demonstrating 6-kb and 10-kb bands in the proband (P), the mother (Mo), and the grandmother (GrMo), and only the 10-kb band in the father (Fa) and the grandfather (GrFa). size, size marker. (**B**) Sanger sequence analysis of the 6-kb bands of the proband and the mother, and the 10-kb band of the father, demonstrating a direct connection between introns 15 and 18 of *ATP7A* (NM_000052.7) in the proband and the mother. (**C**) Relative mRNA expression of exons 16 to 17 of *ATP7A* detected by RT-PCR. Data were normalized to exons 3 to 4 of *ATP7A.* n = 2 technical replicates.

Although short-read sequencing targeted on the *ATP7A* locus detected no variations in the proband, it suggested heterozygous deletion of exons 16 and 17 (Kazusa DNA institution, Japan)^13^. Accordingly, genomic DNA samples of the pedigree were examined. PCR amplification of exons 15 to 18 of *ATP7A* produced 6-kb and 10-kb bands in the proband, the mother, and the grandmother, while only the 10-kb band was produced in the father and the grandfather (Fig. 2A). Subsequent Sanger sequence analysis revealed that the 6-kb PCR product encoded *ATP7A* intron 15 directly connected to intron 18 (NC_000023.10:g.77286595_77290809del), resulting in skipping of exons 16 and 17 in the *ATP7A* cDNA (NM_000052.7) (Fig. 2B), a frameshift at codon 1038 and a premature stop codon at 1054. This variation truncates one-third of the C-terminal domain of ATP7A protein (NP_000043.4). RT-qPCR analysis of peripheral blood leukocytes of the proband, her parents, and her maternal grandmother confirmed almost complete skipping of exons 16 and 17 of the *ATP7A* gene only in the proband (Fig. 2C). Moreover, no pathogenic variants were detected in other genes known to cause copper metabolism disorders including Wilson disease (*ATP7B*)^14^, MEDNIK syndrome (*AP1S1*)^15^, and Huppke-Brendel syndrome (*SLC33A1*)^16^. We concluded that this deletion was the pathogenic variant in the proband.

### Unfavorable switching of skewed XCI in the proband

**Figure 3:**
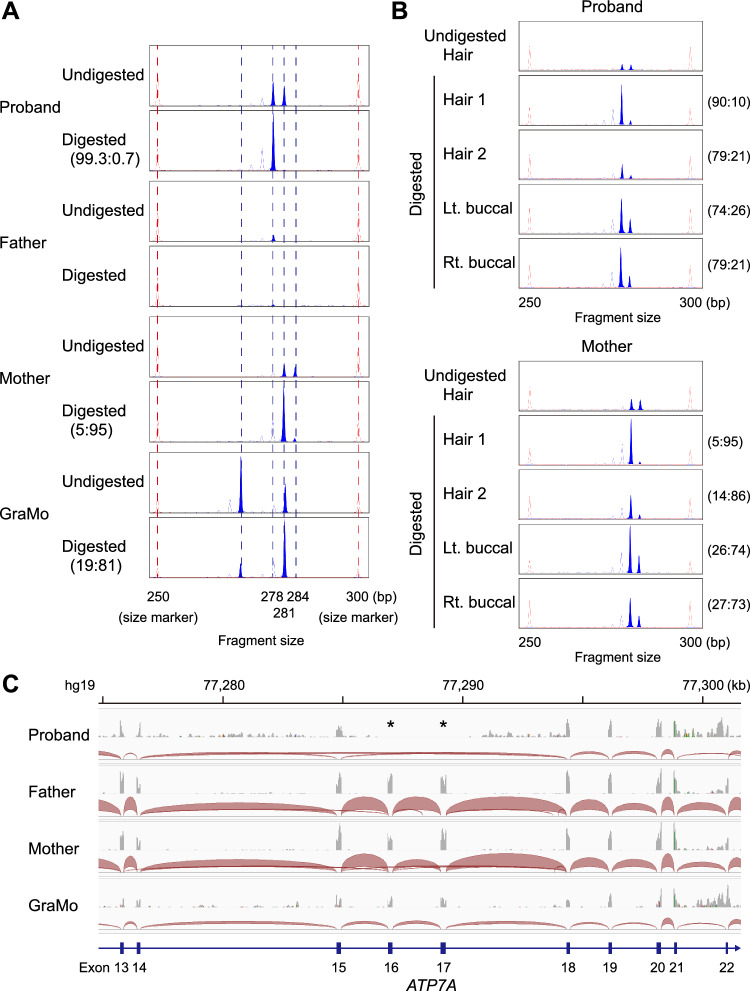
Unfavorable switching of skewed XCI in the proband. (**A**) XCI analysis by methylation-sensitive PCR targeting the *HUMARA* locus. Genomic DNA from peripheral blood of the proband had 278-bp and 281-bp fragments, the former from her father, and the latter from her mother and her grandmother. The inheritance pattern of the 281-bp allele is consistent with the deletion status of the *ATP7A* gene, suggesting that the 281-bp allele represents the deleted *ATP7A* gene allele. While the grandmother and the mother show moderate (81:19) and extreme (95:5), respectively, skewing of XCI toward the 281-bp allele with the *ATP7A* deletion, the proband has extremely skewed XCI (99.3:0.7) toward the normal 278-bp allele. (**B**) XCI analysis of the hair (two samples) and the right (Rt.) and left (Lt.) buccal mucosa of the proband (upper) and the mother (lower), showing a consistent trend of skewed XCI in each individual. (**C**) RNA sequencing of peripheral blood cells of the proband, the father, the mother, and the grandmother (GraMo). The upper grey tracks show coverage depth, and the lower dark red tracks show junctions of RNA sequencing. The proband exclusively expressed the pathogenic *ATP7A* mRNA transcripts lacking exons 16 and 17. Two asterisks indicate no coverage of exons 16 and 17 in the proband.

While heterozygous exon skipping of *ATP7A* was observed in the proband, her mother, and her grandmother, only the proband manifested clinical features of Menkes disease. To understand the mechanism of the development of Menkes disease in the pedigree, the XCI pattern was analyzed by examining the methylation status of highly polymorphic repeat sequences of the X-linked *HUMARA* gene. Genomic DNA obtained from peripheral blood of the proband had 278-bp and 281-bp *HUMARA* alleles. The former was inherited from her father, and the latter from her mother and her grandmother. The inheritance pattern of the 281-bp allele was consistent with the deletion status of the *ATP7A* gene in this pedigree. These findings suggested that the 281-bp allele represented the deleted *ATP7A* gene allele with no recombination between the *HUMARA* gene and the *ATP7A* gene loci. Intriguingly, while the grandmother and the mother showed moderate (81:19) and extreme (95:5), respectively, skewing of XCI toward the 281-bp allele with the *ATP7A* deletion, the proband had extremely skewed XCI (99.3:0.7) toward the normal 278-bp allele (Fig. 3A). Analysis of the hair and buccal mucosa in the proband and the mother indicated consistency of XCI skewing patterns among blood, hair and buccal mucosa (Fig. 3B).

Subsequent RNA sequencing confirmed that the proband exclusively expressed the pathogenic *ATP7A* mRNA transcripts lacking exons 16 and 17 (Fig. 3B). Her mother mainly expressed the normally spliced form of *ATP7A* and only partly expressed transcripts from the pathogenic allele, and her father expressed only normally spliced *ATP7A*. Her grandmother expressed only normally spliced *ATP7A* mRNA, probably due to the shallow depth of RNA sequencing. Taken together, these results revealed that unfavorable switching of XCI skewing toward the normal *ATP7A* allele was the cause of Menkes disease of the proband.

### No deleterious variants on the X chromosome detected by WGS

In humans, mutations in the *XIST* promoter were reported to cause familial XCI skewing. To explore the reason for multiple XCI skewing in this family, WGS was conducted. WGS analysis reconfirmed the *ATP7A* gene deletion from introns 15 to 17 in the proband, her mother, and her grandmother, supporting the PCR-based sequence analysis. On the other hand, no deleterious variants were detected in the lncRNA genes, *XIST, TSIX, JPX,* and *FTX*, in the X inactivation center (XIC). Furthermore, after a thorough bioinformatic and literature search, we were unable to find any other deleterious variants that might affect the XCI process, either inherited or de novo, on the X chromosome in this family.

### Expression skewing across the X chromosome and recombination mapping

**Figure 4:**
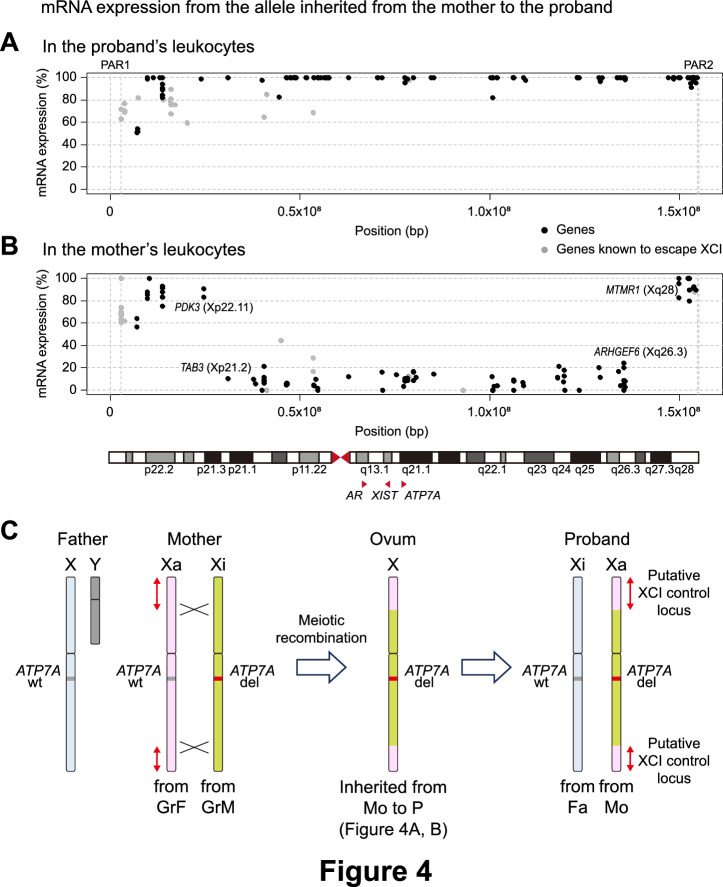
Expression skewing across the X chromosome and recombination mapping. (**A**,**B**) mRNA expression bias of the X chromosome inherited from the mother to the proband analyzed by comparing heterozygous SNPs detected both by WGS and RNA sequencing. Each black dot represents each informative heterozygous SNP. The grey dots are SNPs of genes known to escape XCI. (A) SNPs in the proband demonstrate that most genes across the entire X chromosome were expressed almost exclusively from the maternal allele. (**B**) SNPs in the mother revealed that most genes inside the central region from the short arm (p21.2) to the long arm (q26.3) were suppressed and that genes outside the central region were highly expressed from the inherited allele in the mother’s leukocytes. Because XCI of the mother’s leukocytes was extremely skewed, the points where expression bias patterns were inverted are regarded as the positions of meiotic recombination. The lower panel shows the X chromosome and the red triangles indicate the locations of *AR* at Xq12, *XIST* at Xq13.2, and *ATP7A* at Xq21.1. PAR, pseudoautosomal region. (**C**) A schematic diagram depicting the inheritance pattern of this family. The deleted *ATP7A* locus (del, red) is received from her grandmother (GrM) and her mother (Mo) to the proband (P) and inactivated in the mother, but activated in the proband. The central chromosomal region of the proband was from her mother’s inactivated X chromosome (Xi, light green) derived from her grandmother, and two other distal regions (red arrows, putative XCI control loci) were from her mother’s activated X chromosome (pink) derived from her grandfather (GrF). The X chromosome from the father (Fa) is shown in light blue. wt, wild-type.

Apart from established evidence of the involvement of the XIC locus in XCI^5,6,11,17^, other loci on the X chromosome were also suggested to be related to familial XCI skewing^18,19^. To elucidate the mechanism of switching of XCI skewing between the proband and her mother, mRNA expression of the X chromosome inherited from the mother to the proband was analyzed by comparing heterozygous SNPs detected both by WGS and RNA sequencing. Heterozygous SNPs in the proband demonstrated that most genes across the entire X chromosome, except for genes known to escape XCI^20^, were expressed almost exclusively from the maternal allele, consistent with the findings of XCI assays (Fig. 4A). Expression bias of genes on the inherited allele in her mother’s leukocytes showed a more complicated pattern. While most genes inside the central region from the short arm (p21.2) to the long arm (q26.3), including the deleted *ATP7A* gene, were suppressed, genes outside the central region were highly expressed from the inherited allele in the mother’s leukocytes (Fig. 4B). Because the deleted *ATP7A* locus was received from her grandmother and her mother to the proband and inactivated in the mother, these findings indicated that the central chromosomal region of the proband was from her mother’s inactivated X chromosome derived from her grandmother, and two other distal regions were from her mother’s activated X chromosome derived from her grandfather (Fig. 4C). The inheritance pattern was corroborated by analyzing several heterozygous SNPs around two recombination points in this family (Supplementary Table 2). To hypothesize that a *cis*-acting genetic factor on the X chromosome enhances or suppresses XCI skewing of its allele, meiotic recombination must have occurred between the putative XCI-controlling locus and the *ATP7A* locus. Thus, the factor must be on either of the two distal regions derived from her grandfather.

## Discussion

We diagnosed a female infant with Menkes disease and identified heterozygous deletion of exons 16 and 17 of *ATP7A*, with a familial skewing of XCI. ATP7A has 6 copper binding sites, 8 transmembrane domains, an activation domain, a phosphorylation domain, and a nucleotide domain^3^. The exon 16 encodes the phosphorylation domain and the following exons encode a nucleotide domain and two transmembrane domains. Considering that many missense and frameshift mutations were previously reported in these domains, their ablation by the pathogenic variant of the proband is expected to be deleterious to the function of ATP7A protein. While kinky hair is uniformly found in males with classic Menkes disease, it is found only in about half of heterozygous females with Menkes disease^4^. Thus, the absence of kinky hair does not exclude the diagnosis of Menkes disease.

While heterozygous deletion of the *ATP7A* gene was inherited from her grandmother to her mother and the proband, only the proband suffered from Menkes disease clinically. XCI analysis and expression bias analysis by RNA sequencing revealed that the grandmother and the mother showed XCI skewing toward the pathogenic allele and that most *ATP7A* mRNA transcripts were normal. qPCR analysis showed mRNA transcripts with exons 16 to 17 skipped were less than expected from XCI analysis in the mother and the grandmother (Fig. 2C). This may be due to nonsense-mediated mRNA decay, a translation-coupled mechanism that eliminates potentially harmful mRNAs containing premature stop codons^21^, consistent with the absence of clinical manifestations in them. In stark contrast, the proband had extremely skewed XCI toward the normal allele, resulting in exclusive expression of the pathogenic *ATP7A* mRNA.

Menkes disease occurs in approximately one in 300,000 births^1^. The majority of patients are males, and female carriers are usually asymptomatic. However, at least 22 female patients have been reported to date (Supplementary Table 3). Some of them were due to X-autosomal translocation^22–26^, mosaic of Turner syndrome^27^, and microdeletion of Xq28^4^, and 13 patients had a normal karyotype^4,10,27,28^. Skewed XCI (ratio < 24:76) toward the normal X chromosome was demonstrated in 4 patients, and among them, 3 patients had family members with skewed XCI toward the opposite mutated allele^10^. Therefore, switching of XCI skewing within one pedigree can occur on rare occasions. Familial accumulation of XCI skewing was also reported in other X-linked recessive diseases^18,29^. However, in these previous cases, XCI analysis was not conducted for the grandmother of the proband. Whether or how switching of XCI skewing occurs in a pedigree with XCI skewing over three generations is an open question.

In general, 3 different mechanisms for skewed XCI have been proposed so far. First, skewed XCI can be observed by chance because human XCI occurs when the embryo is composed of only 6 to 16 cells^20^. Analysis of 1005 phenotypically normal females revealed relatively biased XCI (< 20:80) in 14.2% of adult peripheral blood and 4.9% of newborn cord blood, and markedly biased XCI (< 5:95) in 1.7% and 0.2%, respectively^8^. Among 62 female donors of the Genotype-Tissue Expression project, one donor had completely skewed XCI in all 16 tissues available, but with no X-chromosomal abnormality detected by WGS^30^. Secondly, transgenic and deletional analyses in mice support the notion that genetic defects in the XCI process lead to XCI skewing^5,31^. In very rare cases, mutations in the human *XIST* promoter were reported to cause skewed XCI by changing the affinity of CTCF to the binding motif^11^. Finally, cell growth advantage or disadvantage caused by mutations on the X-chromosome also results in XCI skewing. Three hemophilia A carriers with clinical phenotype due to skewed XCI were found to carry an additional deleterious mutation on their non-hemophilic X chromosomes^12^. Skewed XCI is frequent in female carriers of hypoxanthine–guanine phosphoribosyltransferase (HPRT) deficiency, also known as Lesch-Nyhan syndrome, due to selection against HPRT-deficient erythrocyte precursors^32^. In the second and third mechanisms, XCI skewing can be inherited over multiple generations, and the causative abnormality must be on the inherited X chromosome.

This study was originally based on the hypothesis that both the *ATP7A* deletion and XCI skewing were inherited from her grandmother, her mother to the proband. We assumed the existence of an unknown *cis*-acting genetic factor on the X chromosome which enhanced or escaped inactivation of the allele. However, our expression bias analysis and recombination mapping revealed that it was impossible to explain skewed XCI in this family by one common mechanism. To postulate that a factor on the X chromosome regulates XCI of its allele in this pedigree, it must be on either of the two distal regions from her grandfather. This assumption is inconsistent with the apparent inheritance of skewed XCI from her grandmother. Although skewed XCI was observed in the proband, her mother, and her grandmother, our findings demonstrated that skewed XCI of the grandmother was unlikely to be inherited to the mother. XCI skewing of the grandmother might be caused by chance, or *ATP7A* deletion itself might have a very slight disadvantage for cell growth leading to XCI skewing. Further studies are necessary to unveil the mechanism of switching of familial XCI skewing over three generations.

Unfavorable switching of skewed XCI in a family with skewed XCI over multiple generations is extremely rare, and its relationship with meiotic chromosomal recombination has been poorly explored. Although the precise mechanism of XCI was not elucidated in the present case, accumulation of three-generation genomic analyses of familial XCI skewing may aid in the identification of new loci involved in XCI regulation, and eventually provide clinical opportunities to prevent female X-linked recessive disease.

## Methods

### Nucleotide extraction

Genomic DNA was extracted from peripheral blood leukocytes, hair, and buccal swabs of the proband, her parents, and her maternal grandparents. Hair DNA was isolated from single hairs using ISOHAIR (NIPPON GENE, Tokyo, Japan) following the manufacturer’s instructions. Buccal swab-derived DNA was obtained by rubbing the left and right buccal mucosa about 10 times with cotton swabs, followed by stirring in the DNA extract solution of the Phusion Human Specimen Direct PCR Kit (Thermo Fisher Scientific Japan, Tokyo, Japan). Total RNA was extracted from peripheral blood leukocytes of the proband, her parents, and her maternal grandmother.

### Sequence analysis of the *ATP7A* gene

Exons 15 to 18 of *ATP7A* were amplified by PCR using leukocyte DNA of the proband, her parents, and her maternal grandparents. Direct sequencing of the PCR products was performed by FASMAC (Atsugi, Japan). Primer sequences are listed in Supplementary Table 1.

### XCI analysis

XCI patterns were examined using a methylation-sensitive PCR-based assay targeting the human X-linked androgen receptor gene (*HUMARA*) locus as described previously^33,34^. Genomic DNA digested with methylation-sensitive restriction enzyme *Hpa*II (digested) and undigested DNA (undigested) were used as templates to amplify *Hpa*II sites of the first exon of the *HUMARA* gene that contained the highly polymorphic repeat sequence by PCR using primers flanking the locus (Supplementary Table 1). The inactivation rate was calculated using PeakScanner2 (Thermo Fisher Scientific).

### Whole genome sequencing (WGS) analysis

Paired-end sequencing of the whole genome was performed using a DNABSEQ platform (BGI, Beijing, China). Reads that passed the quality filter step were mapped to the reference human genome sequence (hg19) with the Burrows-Wheeler Alignment tool^35^. After removal of duplicate reads and base quality value correction, variants were called and filtered by variant quality score recalibration using the Genome Analysis Toolkit (GATK; Broad Institute, MA, USA)^36^, and annotated by SnpEff^37^.

### RNA sequencing analysis

The sequencing libraries of the proband, her parents, and her maternal grandmother were constructed using the TruSeq mRNA sample preparation kit (Illumina, San Diego, CA, USA). Next-generation sequencing was performed on a NextSeq500 (Illumina) with 75-base pair (bp) single reads. The FASTQ files were aligned to the human genome (hg19) using TopHat^38^.

### Real time-PCR (RT-PCR)

Total RNA was extracted and reverse transcribed using the PrimeScript II 1st strand cDNA synthesis kit (TAKARA, JAPAN). RT-PCR was performed using KAPA SYBR FAST qPCR Master Mix (Sigma Aldrich, St. Louis, MO, USA) and the ViiA7 Real-Time PCR System (Thermo Fisher Scientific). The expression was normalized to exons 3 to 4 of *ATP7A*. The primers are listed in Supplementary Table 1.

### Expression bias analysis and recombination mapping

Single nucleotide polymorphisms (SNP) on the X chromosome detected by both WGS and RNA sequencing were extracted. SNPs on the pseudoautosomal region or with read coverage less than 10 in RNA sequencing were excluded. Among the remaining SNPs, informative heterozygous SNPs in the proband were used to calculate expression levels of gene loci on the allele received from the mother to the proband in the proband leukocytes. Similarly, informative heterozygous SNPs in the mother were used to determine expression levels of the inherited allele in the mother’s leukocytes. Because XCI of the mother’s leukocytes was extremely skewed, the points where expression bias patterns were inverted were regarded as the positions of meiotic chromosome recombination when the maternal X chromosome was inherited to the proband.

### Supplementary Information

Supplementary Tables.

## Data Availability

All relevant data are included in the article. RNA sequencing and WGS data are not publicly available as these could compromise patient privacy. Specific variant information is available from the corresponding author upon reasonable request.

## References

[1] Vairo FPE (2019). A systematic review and evidence-based guideline for diagnosis and treatment of Menkes disease. Mol. Genet. Metab..

[2] Kim MY (2019). Urological problems in patients with menkes disease. J. Korean Med. Sci..

[3] Fujisawa C, Kodama H, Hiroki T, Akasaka Y, Hamanoue M (2019). ATP7A mutations in 66 Japanese patients with Menkes disease and carrier detection: A gene analysis. Pediatr. Int. Off. J. Jpn. Pediatr. Soc..

[4] Smpokou P (2015). Menkes disease in affected females: The clinical disease spectrum. Am. J. Med. Genet. Part A.

[5] Loda A, Collombet S, Heard E (2022). Gene regulation in time and space during X-chromosome inactivation. Nat. Rev. Mol. Cell Biol..

[6] Markaki Y (2021). Xist nucleates local protein gradients to propagate silencing across the X chromosome. Cell.

[7] Okamoto I (2021). The X chromosome dosage compensation program during the development of cynomolgus monkeys. Science.

[8] Amos-Landgraf JM (2006). X chromosome-inactivation patterns of 1005 phenotypically unaffected females. Am. J. Hum. Genet..

[9] Renault NK (2007). Heritable skewed X-chromosome inactivation leads to haemophilia A expression in heterozygous females. Eur. J. Hum. Genet. EJHG.

[10] Møller LB (2012). Clinical expression of Menkes disease in females with normal karyotype. Orphanet J. Rare Dis..

[11] Pugacheva EM (2005). Familial cases of point mutations in the XIST promoter reveal a correlation between CTCF binding and pre-emptive choices of X chromosome inactivation. Hum. Mol. Genet..

[12] Dardik R (2021). Molecular mechanisms of Skewed X-chromosome inactivation in female hemophilia patients-lessons from wide genome analyses. Int. J. Mol. Sci..

[13] Fujiki R (2018). Assessing the accuracy of variant detection in cost-effective gene panel testing by next-generation sequencing. J. Mol. Diagn. JMD.

[14] Bandmann O, Weiss KH, Kaler SG (2015). Wilson's disease and other neurological copper disorders. Lancet Neurol..

[15] Incecik F, Bisgin A, Yılmaz M (2018). MEDNIK syndrome with a frame shift causing mutation in AP1S1 gene and literature review of the clinical features. Metab. Brain Dis..

[16] Chiplunkar S (2016). Huppke-Brendel syndrome in a 7 months old boy with a novel 2-bp deletion in SLC33A1. Metab. Brain Dis..

[17] Augui S, Nora EP, Heard E (2011). Regulation of X-chromosome inactivation by the X-inactivation centre. Nat. Rev. Genet..

[18] Cau M (2006). A locus for familial skewed X chromosome inactivation maps to chromosome Xq25 in a family with a female manifesting Lowe syndrome. J. Hum. Genet..

[19] Naumova AK (1998). Genetic mapping of X-linked loci involved in skewing of X chromosome inactivation in the human. Eur. J. Hum. Genet. EJHG.

[20] Werner JM, Ballouz S, Hover J, Gillis J (2022). Variability of cross-tissue X-chromosome inactivation characterizes timing of human embryonic lineage specification events. Dev. Cell..

[21] Kurosaki T, Popp MW, Maquat LE (2019). Quality and quantity control of gene expression by nonsense-mediated mRNA decay. Nat. Rev. Mol. Cell Biol..

[22] Sirleto P (2009). Lyonization effects of the t(X;16) translocation on the phenotypic expression in a rare female with Menkes disease. Pediatr. Res..

[23] Abusaad I (1999). Clinical expression of Menkes disease in a girl with X;13 translocation. Am. J. Med. Genet..

[24] Sugio Y (1998). Translocation t(X;21)(q13.3; p11.1) in a girl with Menkes disease. Am. J. Med. Genet..

[25] Beck J, Enders H, Schliephacke M, Buchwald-Saal M, Tümer Z (1994). X;1 translocation in a female Menkes patient: Characterization by fluorescence in situ hybridization. Clin. Genet..

[26] Kapur S, Higgins JV, Delp K, Rogers B (1987). Menkes syndrome in a girl with X-autosome translocation. Am. J. Med. Genet..

[27] Gerdes AM (1990). Clinical expression of Menkes syndrome in females. Clin. Genet..

[28] Burgemeister AL (2015). Menkes disease with discordant phenotype in female monozygotic twins. Am. J. Med. Genet. Part A.

[29] Wang Z (2013). Familial skewed x chromosome inactivation in adrenoleukodystrophy manifesting heterozygotes from a Chinese pedigree. PloS One.

[30] Tukiainen T (2017). Landscape of X chromosome inactivation across human tissues. Nature.

[31] Yin H, Wei C, Lee JT (2021). Revisiting the consequences of deleting the X inactivation center. Proc. Natl. Acad. Sci. USA.

[32] Torres RJ, Puig JG (2017). Skewed X inactivation in Lesch-Nyhan disease carrier females. J. Hum. Genet..

[33] Allen RC, Zoghbi HY, Moseley AB, Rosenblatt HM, Belmont JW (1992). Methylation of HpaII and HhaI sites near the polymorphic CAG repeat in the human androgen-receptor gene correlates with X chromosome inactivation. Am. J. Hum. Genet..

[34] Boudewijns M, van Dongen JJ, Langerak AW (2007). The human androgen receptor X-chromosome inactivation assay for clonality diagnostics of natural killer cell proliferations. J. Mol. Diagn. JMD..

[35] Li H, Durbin R (2009). Fast and accurate short read alignment with Burrows–Wheeler transform. Bioinformatics (Oxford, England).

[36] McKenna A (2010). The genome analysis toolkit: A MapReduce framework for analyzing next-generation DNA sequencing data. Genome Res..

[37] Cingolani, P. Variant Annotation and Functional Prediction: SnpEff. *Methods in molecular biology (Clifton, N.J.)*. **2493**, 289–314 (2022). 10.1007/978-1-0716-2293-3_19 35751823

[38] Trapnell C (2012). Differential gene and transcript expression analysis of RNA-seq experiments with TopHat and Cufflinks. Nat. Protocols.

