# Supplementary material for: Unfavorable switching of skewed X chromosome inactivation leads to Menkes disease in a female infant
Source: Sci Rep. 2024 Jan 3;14:440. doi: 10.1038/s41598-023-50668-2 (PMC10764769; doi:10.1038/s41598-023-50668-2)
Supplement: Supplementary file 1 — Supplementary Tables. [file 41598_2023_50668_MOESM1_ESM.doc]

SUPPLEMENTARY INFORMATION

**Unfavorable switching of skewed X chromosome inactivation leads to Menkes disease in a female infant**

Ayumi Matsumoto, et al.

**Supplementary Table 1. Primers used in the current study.**

Name Sequence

*ATP7A* exon 15 F TCCAACTGCTGTGATGGTGGGTACA

*ATP7A* exon 15 R TAATACAGCAGTCCGACCTTTTCT

*HUMARA* F GCTGTGAAGGTTGCTGTTCCTCAT

*HUMARA* R FAM-TCCAGAATCTGTTCCAGAGCGTGC

*ATP7A* exon 3 F CGTTGAGAGGAGCAATAGAAG

*ATP7A* exon 4 R CGATGAAGGCTGAGCTATTAC

*ATP7A* exon 16 F CCCTCTAGGAACAGCCATAACC

*ATP7A* exon 17 R ATACCACAGCCTGGCACAACCT

FAM, Fluorescein Amidites

**Supplementary Table 2. Representative heterozygous SNPs to confirm the inheritance pattern in this pedigree.**

dbSNP Position GrF GrM Father Mother Proband Inheritance

rs2106705 p22.2 C TT T CT CT GrF > M > P

rs143017839 p22.2 G AA A AG AG GrF > M > P

rs2306530 p21.2 T TA T TA TA GrM > M > P

rs3810693 p11.4 C CG C CG CG GrM > M > P

rs2071706 q26.1 G GA G GA GA GrM > M > P

rs41312580 q26.3 G GA G GA GA GrM > M > P

rs9866 q28 A CC C CA CA GrF > M > P

rs5925425 q28 G CC C CG CG GrF > M > P

GrF, grandfather. GrM, grandmother. M, mother. P, proband. The inherited SNPs are underlined.

**Supplementary Table 3. Female Menkes disease patients in the literature.**

Karyotype *ATP7A* alteration XCI (altered:normal) Age, Phenotype

Present case 46,XX Ex16-17del (0.7:99.3) 7 m, severe ID, epilepsy, hypotonia

Matsubara et al.1978 unknown 24 m, autopsy case

Kapur et al. 1987 46,X,t(X;2)(q13;q32.2) N.A., epilepsy, hypotonia

Gerdes et al. 1990 45,X/46,XX 2.5 y, death, ID, epilepsy

Gerdes et al. 1990 46,XX 5 y, ID, epilepsy, hypotonia

Gerdes et al. 1990 46,XX 14 y, ID, epilepsy, hypotonia

Beck et al.1994 46,X,t(X;1)(q13;q12) N.A., hypotonia

Sugio et al. 1998 46,X,t(X;21)(q13.3;p11.1) epilepsy, hypotonia

Abusaad et al. 1999 46,X,t(X;13)(q13.3;q14.3) 18 m, death

Sirleto et al. 2009 46,X,t(X;16)(q13.3;p11.2) 6.5 m, ID, epilepsy, hypotonia

Moller et al. 2012, case1 46,XX c.1946+5G>A (24:76) 5 y, ID, epilepsy, hypotonia

Moller et al. 2012, case2 46,XX unknown (0:100) 34 y, unable to read, write, talk, epilepsy

hypotonia

Moller et al. 2012, case3 46,XX Ex6 del inconclusive 9 y, no language, hypotonia

Moller et al. 2012, case4 46,XX Ex6-9del (80:20) 22 y, mild ID

Moller et al. 2012, case5 46,XX c.4123+5G>A (51:49) 10 y, learning difficulties

Moller et al. 2012, case6 46,XX c.2179G>A, p.G727R (4:96) 2 y, severe ID, hypotonia

Moller et al. 2012, case7 46,XX c.2383C>A, p.R795X (0:100) 41 y, severe ID, epilepsy

Moller et al. 2012, case8 46,XX Ex1del inconclusive 14 y, mild ID

Moller et al. 2012, case9 46,XX c.532G>T, p.E178X (100:0) 29 y, mild ID

Smpokou et al. 2014, case1 Xq28 deletion c.3445del C 22 m, ID, hypotonia, gastrostomy tube

Smpokou et al. 2014, case2 46,XX^(1)^ Ex8-12del 4 y, marked ID, epilepsy, hypotonia,

gastrostomy tube

Smpokou et al. 2014, case3 unknown unknown 7 y, severe ID, epilepsy, hypotonia

Burgemeister, et al. 2015 46,XX^(2)^ Ex6 del* normal* 3 y, death, ID, hypotonia,

gastrostomy tube

ID, intellectual disability. N.A., data not available. y, years of age. m, months of age.

*The healthy monozygotic twin has the same genotype. (1),(2) 46,XX was expected by SNP array CGH or fluorescence in situ hybridization (FISH) with X chromosome-specific DNA-probes, respectively.
